# Estrogen receptor-β expression and pharmacological targeting in bladder cancer

**DOI:** 10.3892/or.2013.2416

**Published:** 2013-04-23

**Authors:** ERIC C. KAUFFMAN, BRIAN D. ROBINSON, MARTIN DOWNES, KATARZYNA MARCINKIEWICZ, SRINIVAS VOURGANTI, DOUGLAS S. SCHERR, LORRAINE J. GUDAS, NIGEL P. MONGAN

**Affiliations:** 1 Department of Urology, Weill Cornell Medical College, New York, NY 10065, USA; 2 Departments of Urology and Cancer Genetics, Roswell Park Cancer Institute, Buffalo, NY 14263, USA; 3 Department of Pathology and Laboratory Medicine, Weill Cornell Medical College, New York, NY 10065, USA; 4 Faculty of Medicine and Health Sciences, School of Veterinary Medicine and Sciences, University of Nottingham, Sutton Bonington Campus, Leicestershire LE12 5RD, UK; 5 Department of Pharmacology, Weill Cornell Medical College, New York, NY 10065, USA; 6 Urologic Oncology Branch, National Cancer Institute of the National Institutes of Health, Bethesda, MD 20892, USA

**Keywords:** epigenetic, hormonal, urothelial, transitional cell

## Abstract

A role for estrogen signaling in urothelial carcinoma of the bladder (UCB) is suggested to be associated with more advanced disease with worse outcomes in women. Estrogen receptor β (ERβ) is the predominant receptor in bladder tissues. We aimed to ascertain whether ERβ correlates with clinicopathological predictors of aggressive bladder cancer and worse survival outcomes. ERβ was measured by immunohistochemistry in malignant and adjacent benign bladder tissues in patients (N=72) with UCB who underwent radical cystectomy. ERβ expression was tested for statistical association with clinicopathological variables and patient survival. ERβ expression was determined in bladder cancer cell lines, and the effects of the selective estrogen modulator tamoxifen and the ERβ agonist diarylpropionitrile on cell growth were determined. The ERβ level was significantly higher in malignant vs. benign urothelium (P<0.001) and was strongly associated with aggressive tumor histology characterized by lymphovascular (P=0.008) and perineural (P=0.006) invasion, and clinical histories of pelvic irradiation (P=0.005), hydronephrosis (P=0.022) and no intravesical chemotherapy (P=0.038). All patients with a high (>70%) percentage of ERβ positivity in tissue with >3-month follow-up developed recurrent disease (P=0.009). Higher ERβ level was predictive of worse recurrence-free and overall survival following cystectomy, after adjustment for tumor stage, and remained significantly associated with recurrence-free survival in the multivariable analysis including tumor stage, nodal stage and lymphovascular invasion. Activation of ERβ in bladder cancer cell lines led to significant increases in proliferation, while pharmacological inhibition with tamoxifen blocked cell growth. Our study supports a role for ERβ in aggressive UCB. Pharmacological targeting of ERβ warrants further investigation as a therapeutic strategy in UCB.

## Introduction

Urothelial carcinoma of the bladder (UCB) is the fourth and tenth most common solid malignancy among US men and women, respectively (1). Radical cystectomy achieves a durable cure in most patients with organ-confined, lymph node-negative muscle-invasive or high-risk non-muscle invasive tumors, but a third of patients recur (2,3). Worse oncologic outcomes are linked to surgical histopathology, including higher pT stage, lymphovascular invasion (LVI), lymph node metastasis and surgical margin involvement, as well as preoperative characteristics including female gender, hydronephrosis, pelvic radiation and lack of neoadjuvant chemotherapy (2–13). A role for hormonal factors in bladder carcinogenesis has been suggested by the male predominance (~3:1) of UCB diagnoses in industrialized nations independent of tobacco usage or occupational carcinogen exposure (3). Estrogen plays a role in bladder development and homeostasis (14) and has been implicated in more advanced stage bladder tumors and worse bladder cancer-specific survival among females (5–13,15). Epidemiologic support for a role of estrogen in bladder carcinogenesis includes a 50–60% increase in UCB diagnoses among females with early menopause, bilateral oophorectomy or absence of combined estrogen-progesterone hormonal replacement therapy (16–18). A 2-fold increase in UCB incidence is also observed among nulliparous women, possibly related to unopposed estrogen (16).

The estrogen receptor genes, ERα and ERβ, have differential tissue expression patterns and functions (19). An important role for estrogen signaling is becoming apparent in UCB (20–24). ERβ protein is the predominant ER in bladder, expressed in up to 77% of clinical UCB, whereas ERα expression is infrequent (<5%) (22,25,26). ERβ expression is unrelated to UCB patient gender or age, but has not been studied with regard to other clinical risk factors, such as smoking or radiation. Furthermore, the relationship between ERβ and tumor stage or patient outcomes is disputed (22,25,26), and the relationship of ERβ to histological cancer features such as perineural invasion, concomitant carcinoma *in situ* or LVI has not yet been described. While selective estrogen receptor modulators (SERM) have been shown to inhibit the proliferation of some UCB cell lines and murine xenografts, which ER isoform(s) is being targeted has not been addressed (20–24). Research in preclinical models has generally not interrogated the ERβ isoform specifically, with most studies evaluating a non-specific ERα/ERβ agonist in UCB cell lines expressing both ERα and ERβ.

As ERβ is the predominant estrogen receptor isoform in normal and malignant bladder tissue, our study aimed to better delineate the role of ERβ in bladder carcinogenesis. We quantified ERβ expression in both malignant and benign tissues from UCB patients undergoing cystectomy and tested its association with tumor pathology, including for the first time histological features such as lymphovascular, perineural invasion and concomitant carcinoma *in situ*, in addition to survival outcomes. We also provide the first investigation of ERβ in relation to known clinical risk factors for UCB diagnosis and UCB-specific mortality including smoking history, race, pelvic radiation, hydronephrosis and neoadjuvant chemotherapy. Finally, we evaluated the effect of selective ERβ activation on UCB cell line proliferation with and without SERM treatment. Our findings indicate a crucial role for ERβ in UCB, including a novel stage-independent association with aggressive cancer histology and poor survival outcomes after cystectomy, and suggest clinical utility of ERβ pharmacologic targeting in this disease.

## Materials and methods

### Patients and tissue specimens

Institutional Review Board approval was acquired for this study. Tissue samples (N=129), including urothelial cell carcinomas (n=59) and benign urothelium (n=70), were obtained from May 2002 to December 2007 at the Department of Pathology and Laboratory Medicine, New York Presbyterian Hospital, Weill Cornell Medical College as previously described (27). All patients had preoperatively diagnosed UCB and underwent pelvic lymphadenectomy during cystectomy. Thirteen patients with no detectable cancer (pT0) at surgery had only benign urothelium acquired. All but two patients without pT0 disease had both benign and malignant tissue samples obtained; the two remaining patients had large primary tumors such that only malignant tissue was available. Death certificates and autopsy reports were reviewed to determine cause of death as cancer-related or from other causes.

### Immunohistochemistry

Immunohistochemical staining for human ERβ protein (14C8 antibody, 1:300 dilution; Novus Biologicals, Littleton, CO, USA) was performed using the Leica BOND-MAX Autostainer and the Leica Microsystems Refine Detection kit as previously described (27). Negative controls were performed without the primary antibody and with normal mouse serum. Benign and malignant prostate tissues were used as a positive control. All stained tissue sections were evaluated by a urologic pathologist (B.D.R.), and separate scores were assigned to each sample based on: i) the percentage of tissue staining positive (0–100%); and ii) intensity of positively staining cells (none, 0; weak, 1; moderate, 2; and strong, 3) as previously described (27). Only nuclear staining was considered positive, and a minimum of 100 tumor nuclei was evaluated per case.

### Bladder cancer cell lines, culture conditions, RNA extraction and qRT-PCR

The HTB-1 (J82), HTB-5 (TCCSUP) and HT1376 UCB cell lines were derived from high-grade invasive urothelial carcinomas, and the HTB-3 (SCaBER) cell line was derived from an invasive squamous cell carcinoma of the bladder (ATCC, Rockville, MD, USA). RNA was extracted and cDNA was synthesized as previously described (27). Primers for ERα and ERβ were as previously described (22) and HPRT (5′-tgctcgagatgtgatgaagg-3′ and 5′-tcccctgttgactggtcatt-3′) was used as a normalizing control. cDNA from the MCF-7 breast cancer cell line was used as a positive control, and expression of ERα and ERβ was normalized by *HPRT* and compared to the levels detected in MCF-7 using the ΔΔCt qPCR method.

### Effects of ER agonists and a selective ER modulator on bladder cancer cell growth

The effects of 17β-estradiol (E2)(Sigma-Aldrich, St. Louis, MO, USA) and selective ERβ agonist diarylpropionitrile (DPN) (Tocris, Minneapolis, MN, USA), with and without tamoxifen (Sigma Aldrich), were assessed on bladder cancer cell lines at a 10 nM concentration as previously reported (22,28). Cells (1×10^6^/well) were treated with the drugs for 48 h and the number of cells was counted using a Coulter Counter (Beckman-Coulter, Brea, CA, USA). Each treatment condition represents a minimum of 12 data points accrued over 4 separate experiments.

### Statistical analysis

Fisher’s exact test was used to test for statistical association between ERβ immunostaining scores and clinicopathological variables (Table I). The staining percentage was assessed as a categorical variable using a 4-tier scale (0–10%, 11–40%, 41–70% and 71–100%), while staining intensity was assessed using a 0–3 scale, as previously described (27). Patients with a final pathology of primary UCB *in situ* (pTis) were censored from the analysis testing for association with concomitant CIS. The Kaplan-Meier method was used to analyze disease-free, cancer-specific and overall survival curves stratified by ERβ immunostaining scores. Time to recurrence or death was compared between groups using a log-rank test. A multivariable Cox proportional hazards model tested the ability of immunostaining scores to predict survival outcomes after adjusting for pathologic tumor stage. A second Cox proportional hazards model was also constructed which included ERβ staining, pathologic tumor stage, LVI and lymph node stage. For *in vitro* growth assays, two-tailed t-tests were utilized to compare the effects on cellular proliferation of individual drug treatments relative to solvent control and between drug combinations. Statistical analyses were conducted using IBM SPSS Statistics (version 19.0.1.80), Prism (GraphPad Software Inc., La Jolla, CA, USA) or JMP version 8 (SAS Institute Inc., Cary, NC, USA). In all cases, P≤0.05 was considered to indicate a statistically significant result.

## Results

### Association of ERβ immunostaining with bladder cancer histopathology and clinical risk factors

**Figure 1:**
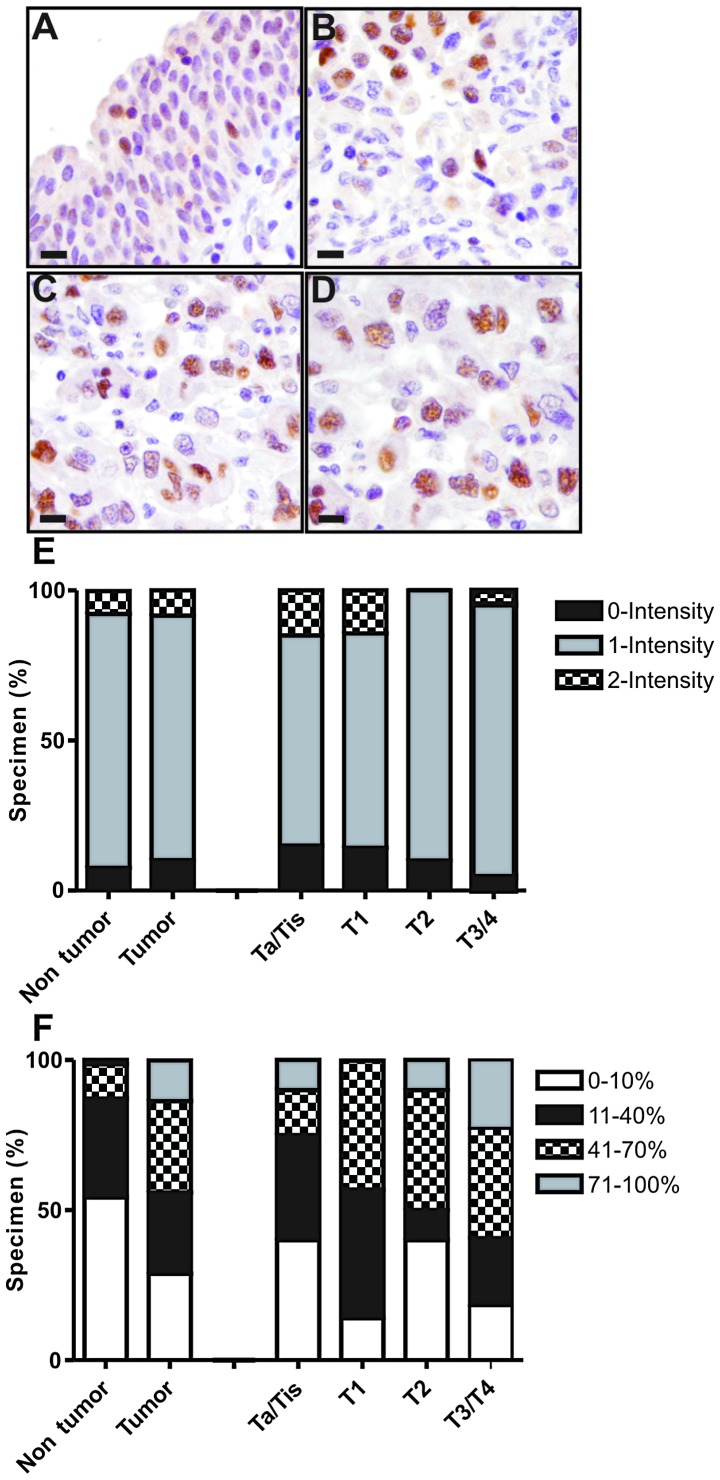
Immunohistochemical staining. Representative immunohistochemical staining for ERβ in (A) benign urothelium, (B) non-muscle invasive urothelial carcinoma (pTis, pTa, pT1), (C) muscle invasive carcinoma (pT2) and (D) extravesical (pT3) carcinoma. (All images were captured at ×400 total magnification; bar, 20 μm). (E and F) Distribution of staining positivity and intensity according to pathological stage. Separate scores were assigned to the stained tissue sections based on (E) the intensity of positively staining cells (0, none; 1, weak; 2, moderate; and 3, strong) and on (F) tissue positivity, i.e., the percentage of tissue staining positive (0–100%). Only nuclear staining was considered positive, and a minimum of 100 tumor nuclei were counted for each case. Please refer to Table II for detailed statistical analysis.

The majority of cases had only low (1+) intensity ERβ staining (Fig. 1). Staining positivity (percentage of tissue) was significantly elevated in cancer tissues compared to benign bladder tissues (P<0.001) (Fig. 1, Table II). For example, ~80% of cancer specimens had >10% tissue positivity, compared to a minority of benign specimens (Fig. 1). High tissue positivity (>70%) was observed exclusively in cancer specimens (Fig. 1). ERβ staining was not associated with tumor stage (Fig. 1), including different substage comparisons (Table II). However, ERβ positivity in cancer specimens was strongly associated with aggressive bladder cancer histological features, namely LVI and perineural invasion (P<0.01 each; Table II). All cancers with LVI had at least low (>10%) ERβ positivity, and all cancers with perineural invasion had at least moderate (>40%) ERβ positivity. The presence of LVI was also associated with ERβ positivity in the adjacent benign urothelium (Table II).

ERβ levels were also tested for association with clinical variables, including risk factors for UCB diagnosis or UCB-specific mortality (Table III). ERβ positivity in cancer specimens was associated with prior pelvic radiation and hydronephrosis (Table III), with >70% positivity found in ~50 and ~33% of cancer specimens with these traits, respectively. ERβ positivity was inversely associated with a history of intravesical chemotherapy, with minimal or low staining in the vast majority (82%) of cancer specimens from these patients. ERβ positivity was associated with race in benign specimens.

### Association of ERβ immunostaining with bladder cancer patient survival outcomes

**Figure 2:**
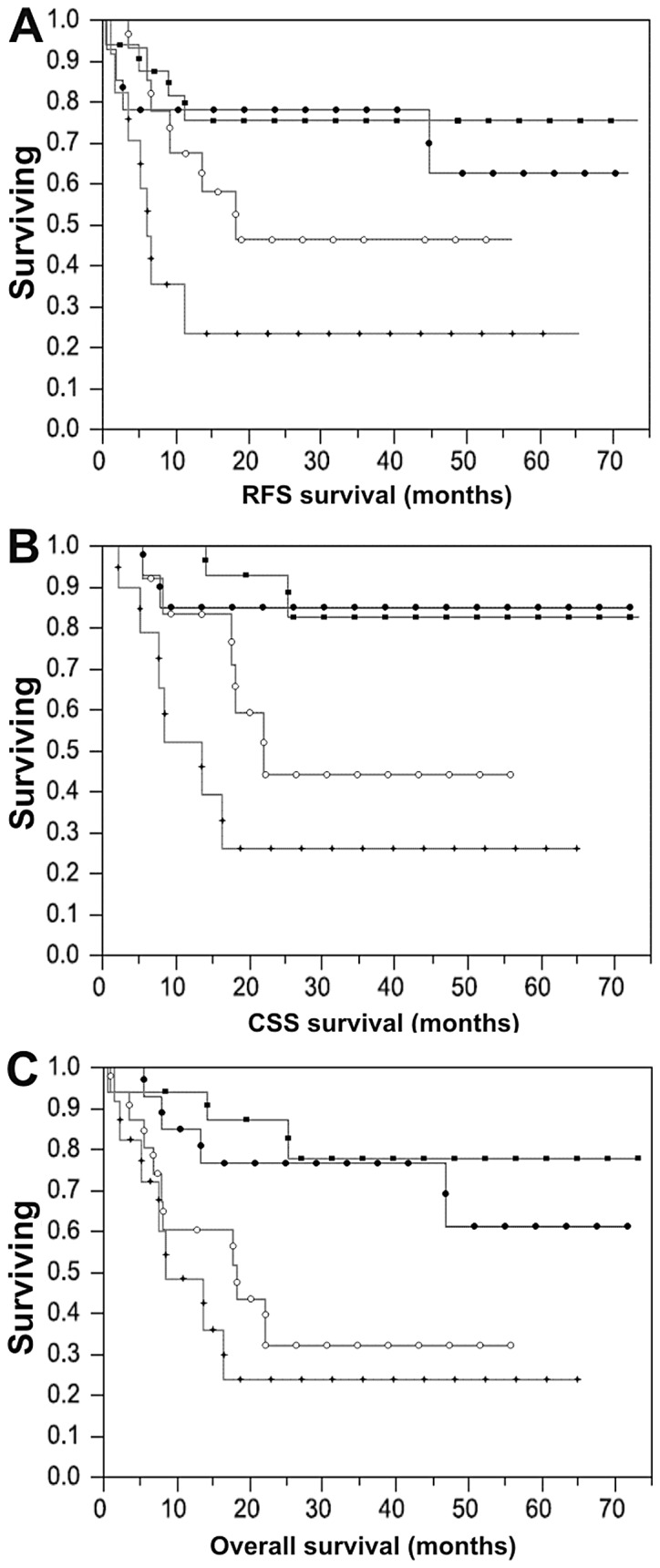
Kaplan-Meier survival estimates. Kaplan-Meier estimates stratified by percentage of ERβ positivity in tissue (square, 0–10%; open circle, 11–40%; black circle, 41–70%; star, 71–100%) for (A) recurrence-free survival (RFS) (P=0.030), (B) cancer-specific survival (CSS) (P=0.0018), and (C) overall survival (P=0.0061).

Median and mean patient follow-up after radical cystectomy was 21 and 26 months, respectively. Recurrence occurred in 21/72 (29%) patients, 15/72 (21%) died from their disease, and an additional 10/72 (14%) patients died of other causes. All patients with a high (>70%) percentage of ERβ positivity in tissue at >3-month follow-up developed recurrent disease. Tumor stage, lymph node stage (P=−0.0001), LVI (P=0.03) and elevated ERβ positivity (P=0.009) were significantly inversely associated with recurrence-free, cancer-specific and overall survival in the Cox univariable analysis (Table IV). Each of these variables also correlated with shorter time to recurrence, cancer-specific or all-cause mortality on log-rank tests, as depicted for ERβ in Fig. 2. No association between ERβ intensity in cancer specimens and time to recurrence, cancer-specific or all-cause mortality was observed (P=0.59, 0.32, 0.54, respectively). There was also no association between ERβ intensity or positivity in adjacent benign urothelium and time to recurrence, cancer-specific or all-cause mortality (P=0.62, 0.08, 0.28 and 0.28, 0.07, 0.18, respectively).

In Cox multivariable analysis including ERβ positivity and tumor stage, both variables remained significantly inversely associated with recurrence-free and overall survival; tumor stage (P=0.02) but not ERβ (P=0.07) remained significantly associated with cancer-specific survival, likely because of the limited number of cancer-specific death events. A second multivariable analysis of survival outcomes was performed with ERβ positivity, tumor stage, lymph node stage and LVI. In this second analysis, increased ERβ positivity still remained significantly inversely associated with recurrence-free survival (P=0.02) independent of these variables (Table IV).

### Effects of ERβ activation and selective ER modulation on bladder cancer cell growth

**Figure 3:**
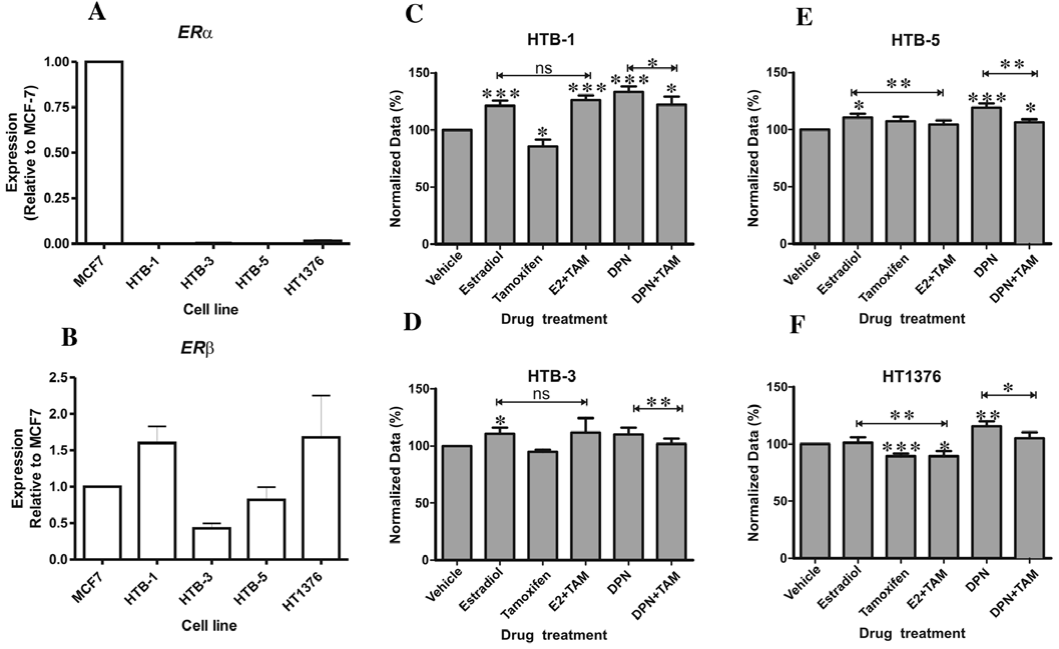
Expression of ERα and ERβ in human bladder cancer cell lines and effects of estrogenic agonists and antagonists on bladder cancer cell proliferation. mRNA expression of (A) ERα and (B) ERβ in bladder cancer cell lines normalized to the expression in MCF7 breast cancer cells. Comparison of the effects of β-estradiol and the ERβ-selective agonist (DPN), in the presence and absence of tamoxifen (TAM) on the proliferation of (C) HTB-1, (D) HTB-3, (E) HTB-5 and (F) HT1376 bladder cancer cells. Statistical significance is indicated relative to control treated cells, except where indicated by bars between treatment groups. (^*^P<0.05, ^**^P<0.01, ^***^P<0.001; ns, not significant).

ERα and ERβ mRNA expression was evaluated in the HTB-1 (J82), HTB-3 (SCaBER), HTB-5 (TCC-SUP) and HT1376 (CRL-1472) bladder cancer cell lines. All four cell lines expressed transcripts for ERβ but not ERα as compared to the MCF-7 breast cancer cell line (Fig. 3A and B). Treatment with estradiol resulted in a significant increase in HTB-1, HTB-3 and HTB-5 proliferation, whereas the selective ER modulator and ERβ antagonist tamoxifen inhibited proliferation of HTB-1 and HT1376 cells, the two cell lines with the highest ERβ expression (Fig. 3B). The ERβ-selective agonist DPN induced a significant increase in proliferation in all three UCB cell lines tested (Fig. 3C, E and F) relative to the control treated cells. DPN did not affect proliferation of the squamous HTB-3 cell line (Fig. 3D), which exhibited the lowest ERβ expression (Fig. 3B). DPN-induced cellular proliferation was inhibited by tamoxifen in all cell lines (Fig. 3). These results indicate that hormonal activation of ERβ promotes UCB cell proliferation and that this can be blocked by tamoxifen.

## Discussion

Identification of the molecular mechanisms governing bladder carcinogenesis is critical for the development of novel pharmacotherapies and biomarkers for this disease. Despite clinical observations of more advanced UCB and shorter survival among female patients, in addition to an established role for ERβ in bladder development and function (14), controversy remains over the contributions of estrogen signaling to bladder carcinogenesis (5–13,15–18). ERβ has been implicated in endocrine-related human malignancies, notably invasive prostate cancers (19). Previous studies of ERα and ERβ expression in UCB specimens yielded inconsistent results (22,25,26,29,30) and have been limited to a subset of available UCB preclinical models (22,28,29).

We sought to clarify these findings by examining ERβ in a well-described UCB patient cohort (27) and additional bladder cancer cell lines. Among the UCB patients, we found no association between ERβ and tumor stage, but uncovered a novel association between elevated ERβ and aggressive bladder histology characterized by perineural invasion and LVI, the latter being a well-established independent predictor of worse oncologic outcomes. In addition, our findings demonstrated that elevated ERβ predicts reduced survival among cystectomy patients with all UCB stages, and provide the first demonstration that this association is independent of tumor stage. ERβ expression remained significantly associated with recurrence-free survival after adjusting for other established independent predictors of cystectomy patient outcomes, including lymph node stage and LVI. We thus identified ERβ as an independent biomarker for poor cystectomy patient outcome, suggesting a model in which estrogen signaling promotes aggressive UCB histology. We additionally found an association between LVI and higher ERβ levels in the adjacent benign urothelium, raising the possibility of paracrine signaling effects in promoting this aggressive UCB histology. We also investigated for the first time the association between ERβ expression and clinical risk factors for UCB diagnosis and/or cancer-specific death. We identify a significant association between elevated ERβ and preoperative hydronephrosis and prior pelvic radiation, both linked to aggressive bladder cancers, suggesting a potential role for ERβ in these processes. Finally, we noted lower ERβ levels in patients who had prior intravesical chemotherapy, consistent with a role of ERβ in promoting cell cycle progression.

To corroborate our patient findings, we determined the effect of ERβ activation on the *in vitro* proliferation of a panel of bladder cancer cell lines. Shen and colleagues (22) previously reported increased proliferation in the RT4 UCB cell line treated with the non-selective ER agonist estradiol; but higher expression of ERα than ERβ in this cell line raises the possibility that ERα activation was responsible. Another study investigating estradiol in a UCB cell line expressing predominantly ERβ observed no growth effects (31). Here, we demonstrated that selective ERβ activation by DPN in the bladder cancer cells predominantly expressing ERβ increased proliferation in all cases (Fig. 3C, E and F), whereas DPN had no positive effect on the proliferation of HTB-3 cells with low ERβ expression (Fig. 3D). These data provide mechanistic evidence that ERβ activation promotes UCB cell growth, although contrasting with a recent report from Han *et al*(29) where ERβ activation reduced UCB migration and invasion.

To explore ERβ as a therapeutic target, we measured the growth effects of the selective ER modulator (SERM), tamoxifen, in UCB cell lines, with or without simultaneous ERβ activation using DPN. Several prior studies of SERMs have described conflicting growth inhibitory effects in bladder cancer cell lines or murine xenografts (21–23,31). We found that tamoxifen treatment alone significantly inhibited growth (Fig. 3C and F) of the two cell lines (HTB-1 and HT1376) with high ERβ expression (Fig. 3B). Tamoxifen also significantly inhibited the proliferative effects of DPN in all 4 bladder cancer cell lines (Fig. 3C-F) when the two treatments were combined, supporting the therapeutic efficacy of selective ER modulators in bladder cancer.

In conclusion, we demonstrated ERβ upregulation in UCB compared to benign urothelium, consistent with an oncogenic function. Furthermore, we uncovered a stage-independent association between ERβ levels and: i) aggressive UCB histology and ii) clinical traits carrying a poor prognosis. We provide the first demonstration of ERβ as an independent predictor of poor cystectomy patient outcomes after adjustment for tumor stage, lymph node involvement and LVI. We demonstrated that ERβ activation increases UCB cell proliferation but can be effectively blocked by SERM pharmacotherapy. Although the relationship between ERβ and tumor stage and patient outcomes is disputed (22,25,26), our research supports a role for ERβ in promoting aggressive bladder cancer and suggests that pharmacological targeting of estrogen signaling pathways may be useful in treating this disease.

## Figures and Tables

**Table I:** Characteristics of the cystectomy patients.

Characteristics	
Total patients, n (%)	72 (100)
Age (years)	
Mean ± SD	66.4±10.0
Median	66.6
Range	43.3–88.8
Gender, n (%)	
Female	21 (29)
Male	51 (71)
Race, n (%)	
Caucasian	66 (92)
Other	6 (8)
BMI, kg/m^2^	
Mean ± SD	26.8±4.8
Median	26.5
Smoking history, n (%)	56 (74)
Active smoker, n (%)	15 (21)
Prior pelvic irradiation, n (%)	8 (11)
Prior intravesical chemotherapy, n (%)	21 (29)
Prior neoadjuvant systemic chemotherapy, n (%)	11 (15)
Pre-operative hydronephrosis, n (%)	20 (28)
Pathological stage, n (%)	
pT0	13 (18)
pTa	9 (13)
pTis	11 (15)
pT1	7 (10)
pT2	10 (15)
pT3	11 (15)
pT4	11 (15)
Lymph node metastases, n (%)	18 (25)
N-stage	
N1	10 (14)
N2	5 (7)
N3	3 (4)
Lymphovascular invasion (LVI), n (%)	15 (21)
Perineural invasion, n (%)	6 (8)
Concomitant carcinoma *in situ* (CIS), n (%)	34 (47)

**Table II:** Association of ERβ with patient histopathological traits, disease recurrence and cancer-specific mortality in tumor specimens.

	Tumor (n=59)		Benign urothelium (n=70)	
		
Histopathological parameters	ERβ positivity %	ERβ intensity	ERβ positivity %	ERβ intensity
Stage (overall)	0.448	0.326	0.499	0.694
Non-muscle invasive vs. invasive	0.197	0.290	0.540	0.288
NMI vs. MI	0.218	0.149	0.425	0.493
BC vs. EVE	0.249	0.457	0.105	0.043
Lymphovascular invasion	**0.008**	0.389	**0.033**	**0.317**
Perineural invasion	**0.006**	1	0.493	1
Comcomitant carcinoma *in situ*	0.230	0.323	0.628	0.823
Positive bladder margin	0.769	1	0.841	1

Fisher’s exact P-values are shown for each statistical association. Bold print indicates significance (P<0.05). Non-muscle invasive (Ta, T1 and CIS) and muscle invasive (T2, T3 and T4) bladder cancers. NMI, non-muscle invasive; MI, muscle invasive; BC, bladder confined; EVE, extravesical.

**Table III:** Association of ERβ with patient clinicopathological traits, disease recurrence and cancer-specific mortality in tumor specimens.

	Tumor (n=59)		Non-tumor (n=70)	
		
Clinical parameters	ERβ % positivity	ERβ intensity	ERβ % positivity	ERβ intensity
Age (years)	0.283	0.630	0.156	0.373
Gender	0.791	1	0.952	0.199
Race	0.183	0.801	**0.018**	0.398
Smoking				
Any history	0.399	0.819	0.813	1
Active	0.478	1	0.441	1
Body mass index	0.198	0.928	0.139	0.677
Prior pelvic radiation	**0.005**	0.310	0.418	0.771
Hydronephrosis	**0.022**	0.122	0.393	1
Intravesical chemotherapy	**0.038**	0.194	0.083	0.330
Neoadjuvant chemotherapy	0.292	0.462	0.701	0.385

Fisher’s exact P-values are shown for each statistical association. Bold print indicates significance (P<0.05).

**Table IV:** Multivariable associations of survival outcomes.

	Univariate analysis	Multivariable analysis 1	Multivariable analysis 2
			
	(P-value)	pT stage (P-value)	pT stage, N stage and LVI (P-value)
Recurrence-free survival			
Percentage of ERβ positivity in tissue	0.0090	0.029	0.017
pT stage	<0.0001	<0.0001	0.0004
N stage	0.0001	-	0.14
Lymphovascular invasion	0.030	-	0.91
Cancer-specific survival			
Percentage of ERβ positivity in tissue	0.0014	0.11	0.26
pT stage	<0.0001	0.016	0.071
N stage	0.0012	-	0.83
Lymphovascular invasion	0.0017	-	0.89
Overall survival			
Percentage of ERβ positivity in tissue	0.0061	0.041	0.067
pT stage	<0.0001	<0.0001	0.017
N stage	0.0003	-	0.58
Lymphovascular invasion	<0.0001	-	0.035

Multivariable analysis 1 includes ERβ percentage and pT stage. Multivariable analysis 2 includes ERβ percentage, pT stage, N stage and lymphovascular invasion (LVI).
